# A Requirement for FGF Signalling in the Formation of Primitive Streak-Like Intermediates from Primitive Ectoderm in Culture

**DOI:** 10.1371/journal.pone.0012555

**Published:** 2010-09-03

**Authors:** Zhiqiang Zheng, Robb U. de Iongh, Peter D. Rathjen, Joy Rathjen

**Affiliations:** 1 Department of Zoology, University of Melbourne, Parkville, Australia; 2 Department of Anatomy and Cell Biology, University of Melbourne, Parkville, Australia; Katholieke Universiteit Leuven, Belgium

## Abstract

**Background:**

Embryonic stem (ES) cells hold considerable promise as a source of cells with therapeutic potential, including cells that can be used for drug screening and in cell replacement therapies. Differentiation of ES cells into the somatic lineages is a regulated process; before the promise of these cells can be realised robust and rational methods for directing differentiation into normal, functional and safe cells need to be developed. Previous *in vivo* studies have implicated fibroblast growth factor (FGF) signalling in lineage specification from pluripotent cells. Although FGF signalling has been suggested as essential for specification of mesoderm and endoderm *in vivo* and in culture, the exact role of this pathway remains unclear.

**Methodology/Principal Findings:**

Using a culture model based on early primitive ectoderm-like (EPL) cells we have investigated the role of FGF signalling in the specification of mesoderm. We were unable to demonstrate any mesoderm inductive capability associated with FGF1, 4 or 8 signalling, even when the factors were present at high concentrations, nor any enhancement in mesoderm formation induced by exogenous BMP4. Furthermore, there was no evidence of alteration of mesoderm sub-type formed with addition of FGF1, 4 or 8. Inhibition of endogenous FGF signalling, however, prevented mesoderm and favoured neural differentiation, suggesting FGF signalling was required but not sufficient for the differentiation of primitive ectoderm into primitive streak-like intermediates. The maintenance of ES cell/early epiblast pluripotent marker expression was also observed in cultures when FGF signalling was inhibited.

**Conclusions/Significance:**

FGF signalling has been shown to be required for the differentiation of primitive ectoderm to neurectoderm. This, coupled with our observations, suggest FGF signalling is required for differentiation of the primitive ectoderm into the germ lineages at gastrulation.

## Introduction

The fibroblast growth factor (FGF) family is a diverse family of growth factors with 22 members identified in humans and mice [1]. FGF signalling has been implicated in mitogenesis [2]–[3], cell migration [4]–[5] and differentiation [6]–[7]. Downstream effectors of FGF signalling include PI3 kinase [8]–[9], Src [10], phospholipase Cγ [9], the MAPK s, Erk (p42/44) [11]–[13] and p38 [9], [14]. A role for FGF signalling in mesoderm formation during embryogenesis was first demonstrated in *Xenopus laevis*; using animal cap assays, it was shown that FGFs induced ventrolateral mesoderm from an unspecified progenitor (reviewed in [15]). Subsequently, roles for FGF signalling in mesoderm formation, maintenance and migration (reviewed in [9]) have been demonstrated during embryonic development of *Drosophila* [16], chick [17], zebrafish [18] and mouse [19]–[21].

Deletion of the FGF receptor, *Fgfr1*, in mice resulted in embryonic lethality before 9.5 d.p.c. [21]. *Fgfr1^−/−^* embryos showed an accumulation of cells adjacent to the primitive streak, defective movement of cells across the primitive streak and flawed axial organisation [21]–[22]. Mutant embryos were also reduced in size compared to heterozygous littermates suggesting a defect in epiblast proliferation. Chimaeric embryos, formed by the injection of *Fgfr1^−/−^* ES cells into wildtype blastocysts, showed accumulation of *Fgfr1^−/−^* cells adjacent to the primitive streak, a deficiency of mutant cells within the nascent mesoderm and formation of ectopic neural tubes comprised exclusively of mutant cells [19]. The ability of *Fgfr1^−/−^* ES cells to form mesoderm in teratocarcinomas suggests that the defect in mesoderm formation during gastrulation is a consequence of abnormal patterning and cell migration and does not reflect a requirement for FGFR1-mediated signalling in determining cell fate [22].

At the time of primitive streak establishment and mesoderm formation in the embryo the expression of *Fgfs 3*, *4*, *5*, and *8* has been detected [23]–[25]. *Fgf4* expression was first detected within the blastocyst [23] where it has been shown to be required for the proliferation of the ICM [26]. Subsequently *Fgf4* transcripts localise to primitive ectoderm adjacent to the primitive streak and to cells within the primitive streak [23]. Disruption of *Fgf4* results in abortive postimplantation development; embryonic lethality occurs prior to primitive streak formation and prevents the analysis of FGF4 function in gastrulation [27]. *Fgf8* transcripts also localise to the primitive streak [28] and are found to be expressed in cells fated to become mesoderm and endoderm cells [26]. Disruption of FGF8 results in a loss of FGF4 expression. In the absence of both FGF4 and FGF8, gastrulation is severely disrupted resulting in a loss of both embryonic mesoderm and endoderm cells [28]. Despite these observations, *in vivo* studies have yet to determine the precise role of FGF signalling in mesoderm specification.

Embryonic stem (ES) cells [29]–[30] have been used extensively to model the events of early mammalian embryogenesis and characterise the roles of developmental regulators. ES cells can be differentiated spontaneously in culture via the formation of embryoid bodies (EBs), which are cellular aggregates formed from ES cells and maintained in medium without the cytokine LIF [31]. Differentiation within EBs results in the formation of a diverse range of cell types including cells representative of the three primary germ lineages and the extraembryonic endoderm [32]–[33]. The addition of an FGF signalling antagonist to EBs demonstrated a requirement for an FGF-mediated epithelial to mesenchymal transition (EMT) during mesoderm specification and again in the establishment of the anterior visceral endoderm [34]. In other differentiation regimes, using modifications of the EB protocol or adherent culture, FGF signalling has been implicated in the formation of the anterior definitive endoderm [35] or maturation of neural stems cells [36] and formation of the neural lineages [34].

ES cells can be differentiated into a uniform population of primitive ectoderm-like cells (early primitive ectoderm-like; EPL) in response to medium conditioned by the human hepatocellular carcinoma cell line HepG2 [37]–[38]. The morphology, differentiation potential, cytokine responsiveness and kinetics of gene expression of EPL cells are consistent with the formation of an *in vitro* equivalent of the embryonic primitive ectoderm, or epiblast [37]–[39]. Primitive ectoderm is an obligatory intermediate in pluripotent cell differentiation *in vivo* and is the immediate progenitor of the germ lineages at gastrulation. Although a primitive ectoderm equivalent forms during ES cell differentiation it is present with other cell populations, most notably cells of the extraembryonic endoderm [38], [40]. These cells act as a source of endogenous signalling and preclude determination of direct or indirect action of exogenously added growth factors. EPL cells form without the concomitant formation of extraembryonic endoderm or other contaminating cell populations, making them an attractive system for the characterisation of growth factor activity during gastrulation. EPL cells have been used successfully to define the roles of cell:cell contact, mesoderm suppression and γ-secretase in lineage choice from pluripotent cells in culture [41]–[42].

In this report we have characterised the role of FGF signalling in EPL cell differentiation and establishment of mesoderm. The use of an FGF signalling inhibitor confirmed a requirement for FGF signalling in the formation of primitive streak-like intermediates from EPL cells and suggests a role for FGF signalling in epiblast maturation. However, the addition of FGFs 1, 4 and/or 8 to EPL cells in culture and in the absence of other inductive activities, was unable to induce the formation of primitive streak-like intermediates even when present at 250 ng/mL. Similarly, we were unable to demonstrate a role for FGFs 4 or 8 in enhancing differentiation induced by BMP4 or altering the sub-type of mesoderm formed in response to BMP4. Together these data suggest that FGF signalling is required for the loss of pluripotence and maturation of primitive ectoderm to a cell able to respond to differentiation-inducing signals, an essential step in establishing the primitive streak and forming primitive streak-like intermediates.

## Results

### EPL cells express *Fgfr1, 2, 3* and *4*

**Figure 1:**
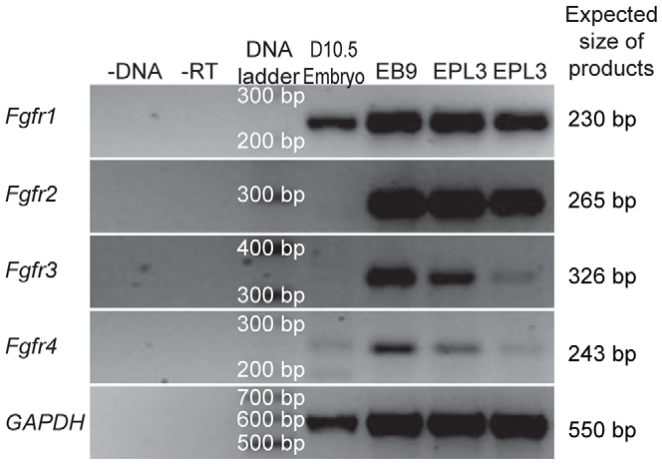
Expression of *Fgfr1-4* in EPL cells. EPL cells (EPL3) were analysed by non-quantitative RT-PCR for the expression of *Fgfr1-4*. Negative controls contained no cDNA (-DNA) or no reverse transcriptase (-RT) was added during cDNA synthesis. Positive controls include 10.5 d.p.c. embryo tissue (D10.5 embryo) and day 9 cell aggregates cultured in serum (D9 ESEB). Expression of *Fgfr1-4* was detected in D9 ESEB and EPL cell aggregates.

FGF signalling is mediated through one of 4 tyrosine kinase receptors, FGFR1-4. The expression of these genes in EPL cells was determined by RT-PCR. EPL cell aggregates were generated by culturing of D3 ES cells in 50% MEDII [38] for 3 days in suspension and analysed using gene specific primers. A band of the expected size (Table 1) was observed for all FGF receptors in day 3 EPL cells (Figure 1). This is consistent with a previous study showing the expression of *Fgfr1* and *Fgfr2* transcripts in the embryonic epiblast [43].

**Table 1:** List of primer sequences, expected amplicon size and number of PCR cycles used.

Gene	Sequence 5′ ->3′	Size of PCR product (bp)	No. of PCR cycles
*β-h1* - F	CAC TGT GAC AAG CTT CAT GTG G	87	26
*β-h1* - R	CCT TGG CAA AAT GAG TAG AAA GG		
*β-major* - F	ACA GGC TGC CTT CCA GAA GG	115	30
*β-major* - R	ACT GAC AGA TGC TCT CTT GGG		
*Sox1* - F	GACTTGCAGGCTATGTACAACATC	171	26
*Sox1* - R	CCTCTCAGACGGTGGAGTTATATT		
*Mash1* - F	TAGCCCAGAGGAACAAGAGC	86	26
*Mash1* - R	CTGCTTCCAAAGTCCATTCC		
*Fgfr1* - F	CAAGAAGAGCGACTTCCATAGCCAGATG	230	35
*Fgfr1* - R	AGACCAGTCTGTCTCGTGGCAGC		
*Fgfr2* - F	CGCCTGTGAGAGAGAGAAGGAGATCACG	265	35
*Fgfr2* - R	GCTGTTGAGGACAGACGCGTTGTT		
*Fgfr3* - F	AAACTGATGAGGCTGGCAGCGTG	326	35
*Fgfr3* - R	GTGTCAGCCGGGTCCTGGATAGC		
*Fgfr4* - F	GTACCCTCGGACCGCGGCACATAC	243	35
*Fgfr4* - R	GCCGAAGCTGCTGCCGTTGATG		
*GAPDH* - F	CTTCACCACCATGGAGAAGGC	236	18
*GAPDH* - R	GGCATGGACTGTGGTCATGAG		
*Brachyury* (*T*) - F	TGCTGCCTGTGAGTCATAAC	143	26
*Brachyury* (*T*) - R	GCCTCGAAAGAACTGAGCTC		
*Wnt3* - F	TCCACTGGTGCTGCTATGTC	138	26
*Wnt3* - R	CCTGCTTCTCATGGGACTTC		
*Bmp4* - F	AGGAGGAGGAGGAAGAGCAG	118	26
*Bmp4* - R	CCTGGGATGTTCTCCAGATG		
*Mixl1* - F	CTTCCGACAGACCATGTACCC	145	30
*Mixl1* - R	GATAAGGGCTGAAATGACTTCCC		
*Flk1* - F	CACCTGGCACTCTCCACCTTC	240	30
*Flk1* - R	GATTTCATCCCACTACCGAAAG		
*Snail1* - F	GCCGGAAGCCCAACTATAGC	64	30
*Snail1* - R	TAGGGCTGCTGGAAGGTGAA		
*Snail2* - F	GCTCCTTCCTGGTCAAGAAACA	117	30
*Snail2* - R	TGACAGGTATAGGGTAACTTTCATAGAGA		
*Cer1* - F	AGAGGTTCTGGCATCGGTTC	229	26
*Cer1* - R	GAGCAGAAGCTGTGGGCATC		
*Chrd* - F	AGACCAAGCCTCAGCGGAAG	155	30
*Chrd* - R	GGTACCAACATTCAGGAACAGC		
*Mesp1* - F	TCGTTCCAGTACGCAGAAACAGC	179	26
*Mesp1* - R	TCAGACAGGGTGACAATCATCCG		
*Fgf4* - F	ACTACCTGCTGGGCCTCAA	169	32
*Fgf4* - R	ACTCCGAAGATGCTCACCAC		
*Fgf8* - F	TCCGGACCTACCAGCTCTAC	143	26
*Fgf8* - R	TCGGACTCTGCTTCCAAAAG		
*Rex1* - F	TGC CTC CAA GTG TTG TCC C	118	qPCR
*Rex1* - R	ATT CAT GTT GTC TTA GCT GCT TCC		
*Fgf5* - F	CTG CAG ATC TAC CCG GAT G	169	qPCR
*Fgf5* - R	TAA ATT TGG CAC TTG CAT GG		
*Oct4* - F	CCC AGG CCG ACG TGG	66	qPCR
*Oct4* - R	GAT GGT GGT CTG GCT GAA CAC		

### FGF signalling is required for the formation of primitive streak-like intermediates from primitive ectoderm

Previous reports have suggested that inhibition of endogenous FGF signalling during ES cell differentiation within EBs results in disruption of the EMT that occurs with mesoderm specification and a reduction in anterior visceral endoderm formation [34]. In this report we differentiated EPL cells in serum-containing medium supplemented with PD173074. PD173074 is a well-characterised competitive inhibitor of FGFR1 that complexes with the kinase domain of the receptor. PD173074 acts to inhibit autophosphorylation of the receptor and receptor kinase activity; the IC_50_ for these activities is 1 to 5 nM and 25 nM respectively [44]–[45]. PD173074 was also reported to inhibit FGFR 2 [45], 3 [45]–[46] & 4 [47].

EPL cells, formed as aggregates, were transferred on day 3 to serum-containing differentiation medium supplemented with 10 nM, 25 nM, 50 nM and 75 nM PD173074 or with 0.00075% DMSO. Aggregates were maintained in this medium for 72 hours, after which they were seeded individually into 2 mL wells containing serum-free medium and allowed to differentiate for a further 6 days. On day 12 adherent aggregates were scored for the presence of beating cardiomyocytes, visible red blood and neural extensions.

**Figure 2:**
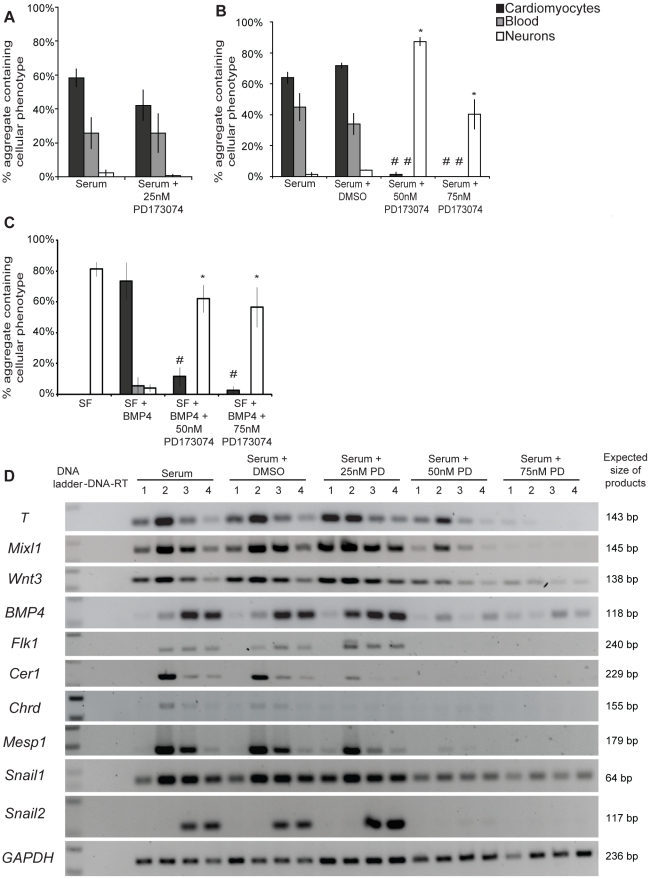
Differentiation outcomes and gene expression from EPL cells cultured in an FGF signalling inhibitor. (**A**) Differentiation outcomes from EPL cells cultured in serum-containing medium supplemented with 25 nM PD173074. n = 7. (**B**) Differentiation outcomes from EPL cells cultured in serum-containing medium supplemented with 50 and 75 nM PD173074. Addition of PD173074 resulted in a significant decrease in cardiomyocytes and blood and a significant increase in neuron formation. n = 3. * indicates a significant increase (p<0.05) when compared to serum + DMSO. # indicates a significant decrease (p<0.05) when compared to serum + DMSO. (**C**) Scored differentiation outcomes of EPL cells cultured in serum-free medium supplemented with 10 ng/ml BMP4 and 50 or 75 nM PD173074. Addition of PD173074 at 50 and 75 nM concentrations had significantly reduced cardiomyocytes (p<0.05) and increased neuron (p<0.05) formation. * indicates a significant increase when compared to serum-free medium containing 10 ng/ml BMP4; p<0.05. # indicates a significant decrease when compared to serum-free medium containing 10 ng/ml BMP4; p<0.05. n = 3. (**D**) Gene expression of EPL cells cultured in serum-containing media supplemented with 0.00025% DMSO, 25, 50 or 75 nM of PD173074 for 1, 2, 3 and 4 days was analysed using RT-PCR. n = 3. A representative result is shown.

Aggregates differentiated in serum-containing medium without PD173074 formed mesoderm efficiently (58–72% forming cardiomyocytes and 25–45% of aggregates forming visible red blood cells; Figure 2A–B) and few neural extensions (<5%; Figure 2A–B). Addition of 10 (data not shown) and 25 nM PD173074 resulted in differentiation outcomes indistinguishable from the control (Figure 2A). In contrast, addition of 50 nM PD173074 resulted in a significant decrease in the number of aggregates containing beating cardiomyocytes and visible red blood cells (1% and 0% respectively, p<0.05; Figure 2B) accompanied by a marked increase in the number of aggregates containing neural extensions (87%, p<0.05; Figure 2B). A similar decrease in mesoderm differentiation was observed in aggregates cultured in 75 nM PD173074. Similarly, reduced mesoderm (cardiomyocytes and visible red blood cells) and increased neural formation was seen when 50 and 75 nM PD173074 was added to aggregates differentiated in serum-free medium with 10 ng/ml BMP4 as the mesoderm inducer (Figure 2C).

**Figure 3:**
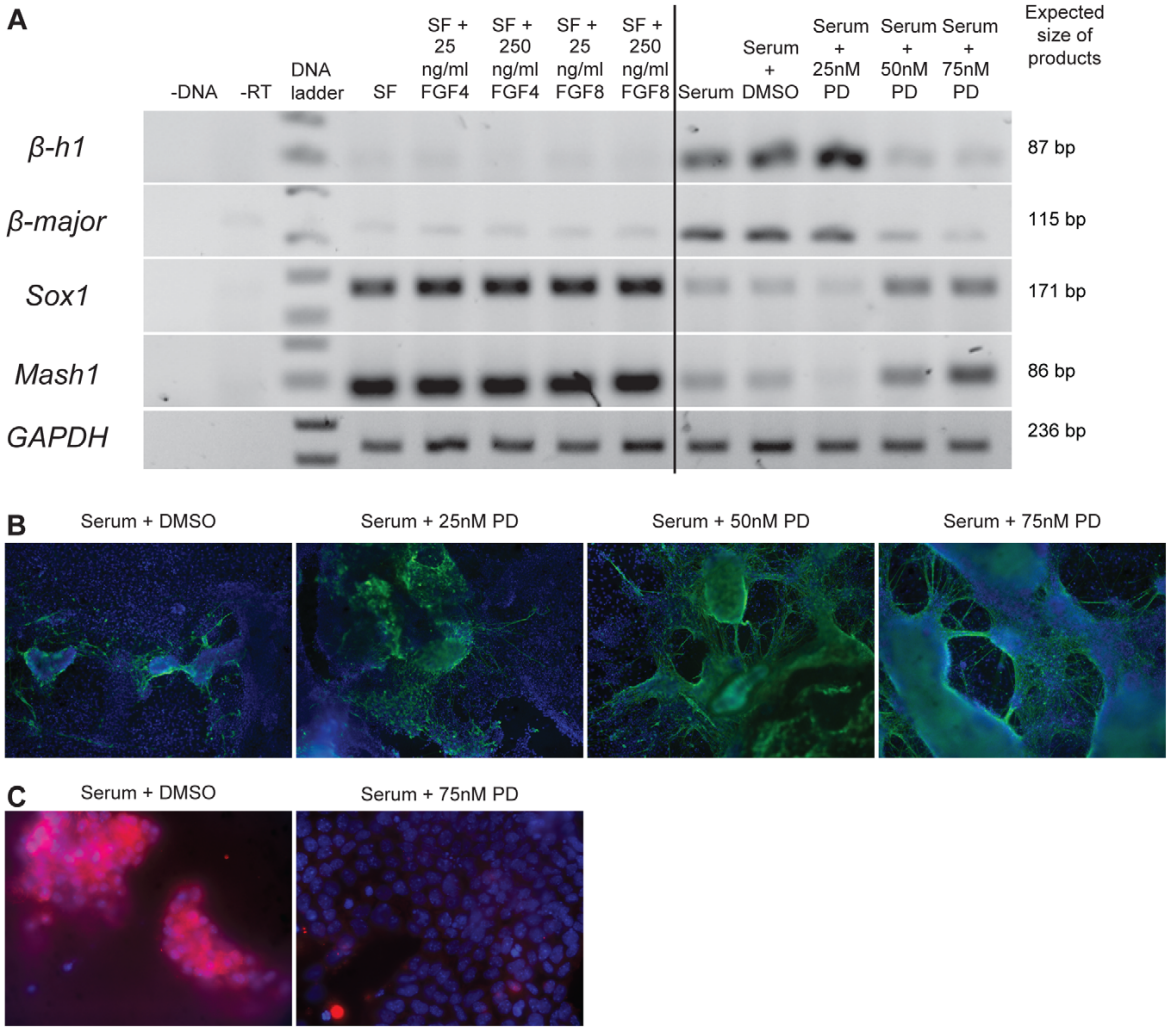
Inhibition of FGF signalling results in increased neurectoderm formation. (**A**) (Left) Gene expression of day 9 cells cultured in serum-free medium supplemented with 25 or 250 ng/ml of FGF4 or 8. (Right) Gene expression of day 9 cells cultured in serum-containing medium supplemented with 25, 50 or 75 nM of PD173074. Treatment of cells with 50 and 75 nM of PD173074 resulted in reduced expression of globin genes (*β-h1*, *β-major*) and increase expression of neural markers (*Sox1*, *Mash1*) as compared to controls. n = 3. A representative result is shown. (**B**) EPL cells were cultured in serum-containing medium supplemented with 25, 50 or 75 nM PD173074 for 4 days and transferred into serum-free medium till day 9. Day 9 cells were stained for βIII-tubulin. Increased βIII-tubulin staining was observed with treatment of PD173074 at 50 and 75 nM concentrations. (**C**) Day 9 cells showed reduced protein expression of cardiac troponin 1 upon treatment of PD173074 at 75 nM. (**B–C**) No fluorescence was detected in the secondary antibody only controls (data not shown).

RNA was extracted from treated aggregates on day 12 and analysed for expression of β-globin genes (*β-h1*, *β-major*) and neural markers (*Sox1*, *Mash1*) via RT-PCR. PD173074 did not appear to affect the expression levels of these markers at 25 nM. At 50 and 75 nM PD173074, however, reduced globin gene expression and increased neural marker gene expression was observed (Figure 3A). Immunofluorescence analysis of these cells with βIII-tubulin and cardiac troponin 1 resulted in similar observations. Increased staining with βIII-tubulin was observed at 50 and 75 nM PD173074 (Figure. 3B) while reduced cardiac troponin 1 staining was observed at 75 nM PD173074 (Figure. 3C).

These data suggested that inhibition of endogenous FGF signalling resulted in a reduction in mesoderm formation. The effect of PD173074 on the formation of primitive streak intermediates and nascent mesoderm was determined by RT-PCR. EPL cells differentiated in the presence of 25, 50 and 75 nM PD173074 were collected after 24, 48, 72 and 96 hours and analysed for expression of primitive streak (*T*, *Mixl1*, *Wnt3*), anterior (*Chrd*, *Cer1*) or posterior (*Mesp1*) streak, mesoderm (*Bmp4, Flk1*) and EMT (*Snail1*, *Snail2*) markers. The presence of 25 nM PD173074 did not prevent the expression of primitive streak and mesoderm markers in differentiating EPL cells. In contrast, we noted a precocious expression of primitive streak markers such as *T*, *Bmp4* and *Mixl1* and a decrease of anterior primitive streak markers *Cer1* and *Chrd* (Figure 2D). Differentiation in 50 and 75 nM PD173074, however, resulted in a marked reduction in the expression of all gene markers, suggesting that commitment to primitive streak intermediate, during early mesoderm formation, is inhibited in the presence of PD173074 (Figure 2D). The reduction in marker expression appeared to be dose dependent.

Collectively, these data indicate a requirement for FGF signalling in the differentiation of the primitive ectoderm to primitive streak intermediates.

### FGFs are unable to induce mesoderm from EPL cells *in vitro*

**Figure 4:**
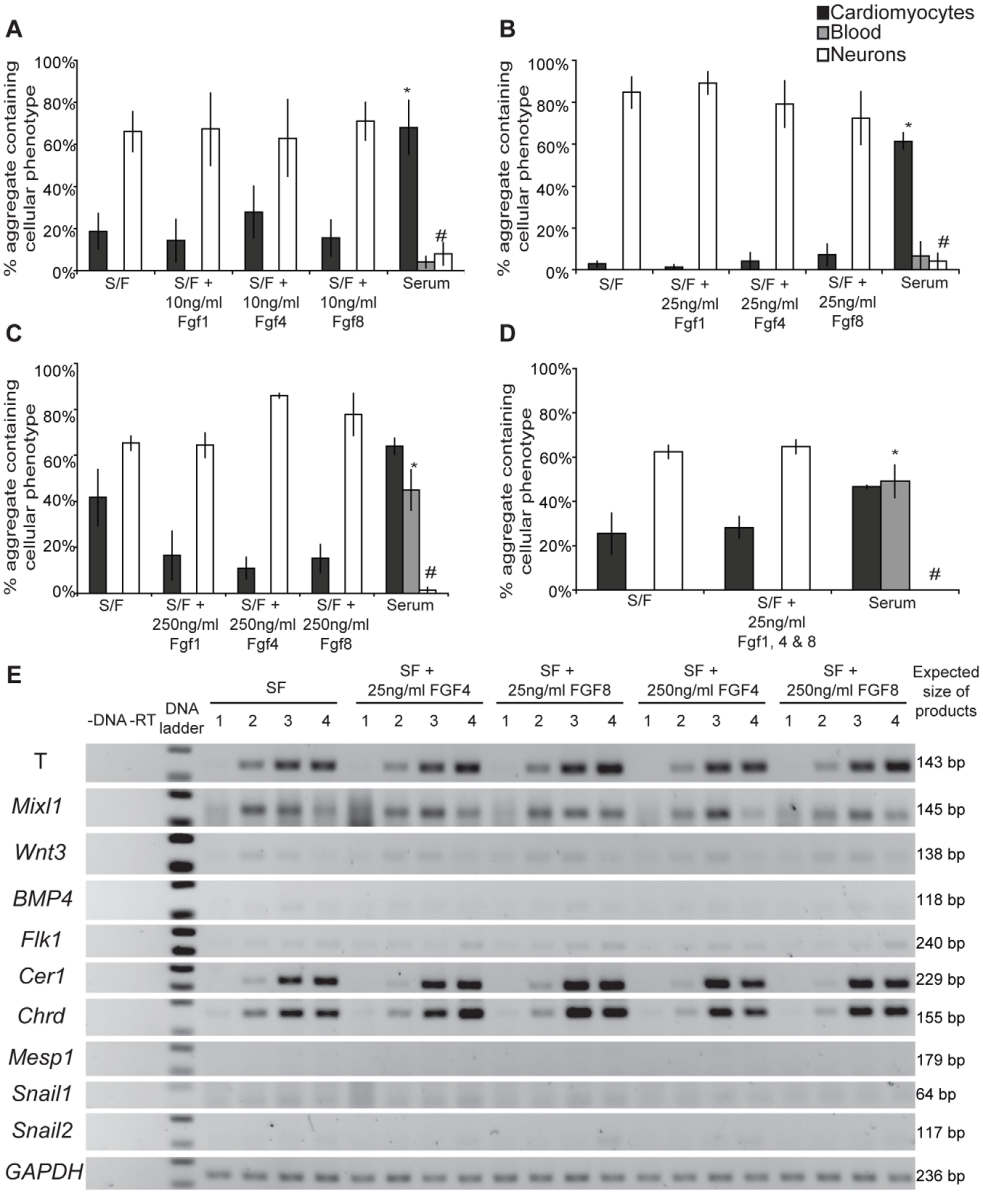
Differentiation outcomes and gene expression of EPL cells cultured in FGFs 1, 4 or 8, or a combination FGFs 1, 4 and 8. (**A–D**) Differentiation outcomes for EPL cells cultured in serum-free medium supplemented with 10, 25 or 250 ng/mL of FGFs 1, 4 or 8, or a combination of FGFs 1, 4 and 8 at 25 ng/mL, are statistically similar to their respective serum-free controls. Serum-containing controls have significantly increased cardiomyocytes or blood formation and significantly lower neuron formation compared to the serum-free controls. * indicates a significant increase (p<0.05) when compared to serum-free medium, # indicates a significant decrease (p<0.05) compared to serum-free medium. n = 3. (**E**) Gene expression of EPL cells cultured in serum-free medium supplemented with 25 or 250 ng/mL of FGFs 4 or 8 for 1, 2, 3 and 4 days was analysed using RT-PCR. n = 3. A representative result is shown.

There is little information on the ability of exogenously added FGFs to exert an effect on pluripotent cell differentiation in culture. This may reflect deficiencies in the assay systems used to date or a lack of inductive capacity associated with this growth factor family. Here, EPL cell aggregates were cultured in serum-free differentiation medium supplemented with 10 ng/mL and 25 ng/mL of FGF1, 4 or 8 for 72 hours. Alternatively, EPL cell aggregates were cultured in serum-free differentiation medium containing a saturating concentration of FGF1, 4 or 8 (250 ng/mL) for 72 hours to ensure that any effect of the factors in this assay would be detected. After treatment, individual aggregates were transferred into 2 mL wells containing unsupplemented serum-free medium, allowed to differentiate and scored for the presence of beating cardiomyocytes, visible red blood and neural extensions (Figure 4A–C). EPL cells maintained in serum-free differentiation medium formed neurons efficiently (65% to 85%) when compared to controls cultured in serum-containing medium (<8%). The formation of beating cardiomyocytes in serum-free aggregates (3% to 30%), however, was significantly lower than serum containing controls (>60%). Also, although red blood cells were readily detected in serum containing controls, no blood formation was detected in aggregates cultured in serum-free differentiation medium. These data suggest that mesoderm is formed inefficiently in aggregates cultured in serum-free differentiation medium; these aggregates provide an ideal assay system for detecting the inductive capacity of FGFs 1, 4 and 8 in culture. Aggregates cultured in FGFs 1, 4 or 8, however, showed differentiation outcomes indistinguishable from controls cultured in serum-free differentiation medium (Figure 4A–C).

In addition to differentiation outcome, EPL cells differentiated in the presence of FGFs 4 and 8 were analysed for the expression of a panel of marker genes of the primitive streak, mesoderm and EMT, as for Figure 2D. As expected from the results of the differentiation assay, gene expression analysis did not show any observable differences in differentiation markers in cells differentiated in the presence of either FGF4 or 8 when compared to controls (Figure 3C&4E).

Within the primitive streak a number of FGF family members are present suggesting the possibility that these factors act synergistically to induce mesoderm formation within this environment. To address this possibility, EPL cell aggregates were cultured in serum-free differentiation medium supplemented with a combination of FGFs 1, 4 and 8 at 25 ng/mL each. The treatment with 25 ng/mL of FGFs 1, 4 and 8 in combination did not yield any significant differences in the formation of beating cardiomyocytes, visible red blood or neural extensions when compared to the serum-free controls. This further supports the notion that the FGFs are unable to induce mesoderm from EPL cells in culture (Figure 4D).

### Can FGF signalling specify nascent mesoderm formed during EPL cell differentiation in culture?

Previous studies in lower vertebrates and insects have suggested that FGF signalling is required for mesoderm induction [48]–[50]. In mouse, it has been suggested these factors act to specify mesoderm and/or endoderm subtypes during gastrulation [34]–[35]. Here, we analysed the ability of FGF signalling to alter the mesoderm subtypes formed from EPL cells induced to form mesoderm in the presence of BMP4 [51]–[52]. BMP4 is a mesoderm inducer that typically enriches the formation of more posterior mesoderm populations, such as primitive blood, during pluripotent cell differentiation [34], [53]–[54]. If FGF signalling was acting to specify nascent mesoderm we might expect to observe changes in the relative frequencies of beating cardiomyocytes or visible red blood cells, or a loss of these outcomes and the formation of a ‘non-recognisable’ mesoderm.

**Figure 5:**
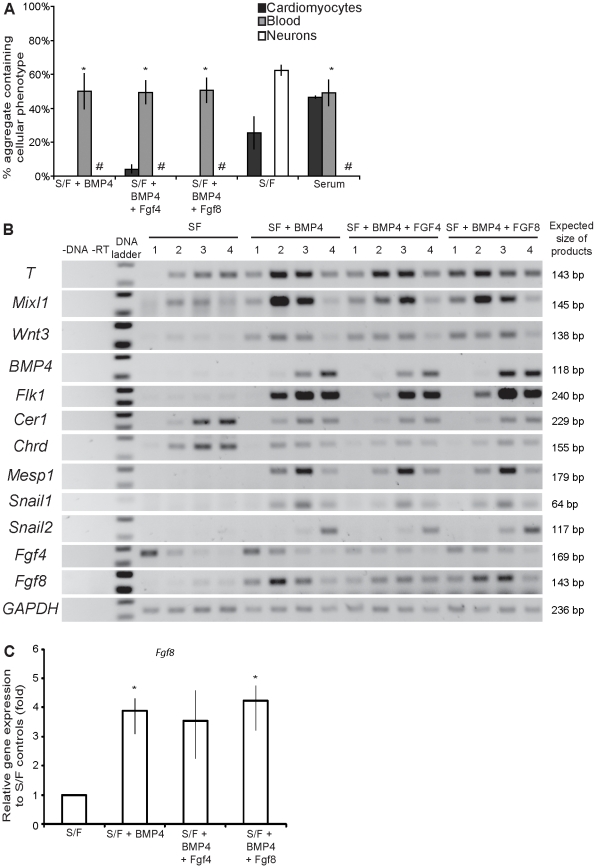
Addition of FGF4 or 8 does not alter the differentiation outcomes and gene expression from EPL cells differentiating in response to BMP4. (**A**) EPL cells were cultured in serum-free medium supplemented with 10 ng/mL BMP4 and 25 ng/mL FGFs 4 or 8 and the outcomes of differentiation scored. Significantly more BMP4-treated EPL cell aggregates formed blood, and fewer formed neural extensions, compared to serum-free controls. Differentiation outcomes of cell aggregates treated with BMP4 and FGF4 or FGF8 were statistically similar to the BMP4-treated controls. * indicates a significant increase when compared to serum-free medium; p<0.05. # indicates a significant decrease when compared to serum-free medium; p<0.05. n = 3. (**B**) Gene expression of EPL cells cultured in serum-free medium supplemented with BMP4 and FGFs 4 or 8 for 1, 2, 3 and 4 days was analysed using RT-PCR. EPL cells cultured in BMP4 showed increased gene expression of primitive streak and mesoderm markers (*T*, *Mixl1*, *Wnt3*, *Bmp4*, *Flk1*), anterior (*Cer1*, *Chrd*) and posterior (*Mesp1*) streak markers, EMT (*Snail1*, *Snail2*). No changes in gene expression were observed with the further addition of 25 ng/mL of FGFs 4 or 8. n = 3. A representative result is shown. (**C**) EPL cells differentiated as for (B) for 2 days were analysed by qRT-PCR for the expression of *Fgf8*. *Fgf8* expression was significantly increased with addition of BMP4 when compared to serum-free controls where p<0.05. All data were analysed relative to GAPDH and normalised to S/F controls. Error bars represent SEM. n = 3.

EPL cell aggregates were cultured in serum-free differentiation medium supplemented with 10 ng/mL of BMP4 and 25 ng/mL of FGF4 or 8 for 72–96 hours. As for Figures 2 and 4, individual aggregates were transferred into 2 mL wells containing unsupplemented serum-free medium and scored for observable differentiation outcomes. The treatment of EPL cell aggregates with BMP4 resulted in morphological changes in aggregate structure; in comparison to controls these aggregates appeared to be loosely packed with ruffled edges (data not shown). This morphology was not altered by the addition of 25 ng/mL of FGFs 4 or 8. As before, aggregates cultured in serum-free differentiation medium formed neurons preferentially, with 62% of these aggregates forming neural extensions and 26% forming beating cardiomyocytes; the addition of FGF 4 or 8 did not alter the overall prevalence of these differentiation outcomes. In the presence of BMP4, 50% of aggregates contained visible red blood cells and no aggregates formed neural extensions. Again, no significant differences in differentiation outcomes was observed between aggregates cultured in BMP4 and FGFs 4 or 8 as compared to those cultured in BMP4 alone (Figure 5A).

We did observe variability in the mesoderm populations induced by BMP4 during the course of this work, as illustrated by the different outcomes seen in Figures 2C and 5A; this is potentially related to the use of different batches of BMP4 throughout these experiments. Regardless of the subtle changes in mesoderm produced, a consistent induction of mesoderm and reduced ectoderm formation was observed upon BMP4 treatment. Interpretation of our data was not affected as mesoderm outcomes were analysed collectively.

EPL cell aggregates cultured in serum-free differentiation medium supplemented with BMP4 and FGFs 4 and 8 were analysed for the expression of a panel of markers of the primitive streak, mesoderm and EMT, as for Figure 5B. EPL cell aggregates differentiated in the presence of BMP4 showed a robust upregulation of primitive streak (*T*, *Wnt3*, *Mixl1*), mesoderm (*Bmp4, Flk1*) and EMT (*Snail1*, *Snail2*) markers. Cells differentiated in BMP4 showed low expression of anterior streak genes (*Cer1*, *Chrd*) and detectable expression of the posterior streak marker *Mesp1*, suggesting the enrichment of more posterior mesoderm populations. The addition of FGFs 4 or 8 to BMP4 treated cultures did not alter the gene expression pattern (Figure 5B–C), suggesting that FGF signalling was unable to alter mesoderm specification during the differentiation of EPL cells to mesoderm. Potentially, BMP4 induces the expression of one or more of the FGF family members during mesoderm induction. qRT-PCR analyses of EPL cell aggregates treated with BMP4 and FGFs 4 or 8 for 2 days revealed a significant upregulation of the *Fgf8* transcripts in the aggregates cultured in BMP4 (Figure 5C). No equivalent upregulation of *Fgf4* was seen (Figure 5B, data not shown).

### FGF signalling affects ES cell pluripotency and epiblast maturation

**Figure 6:**
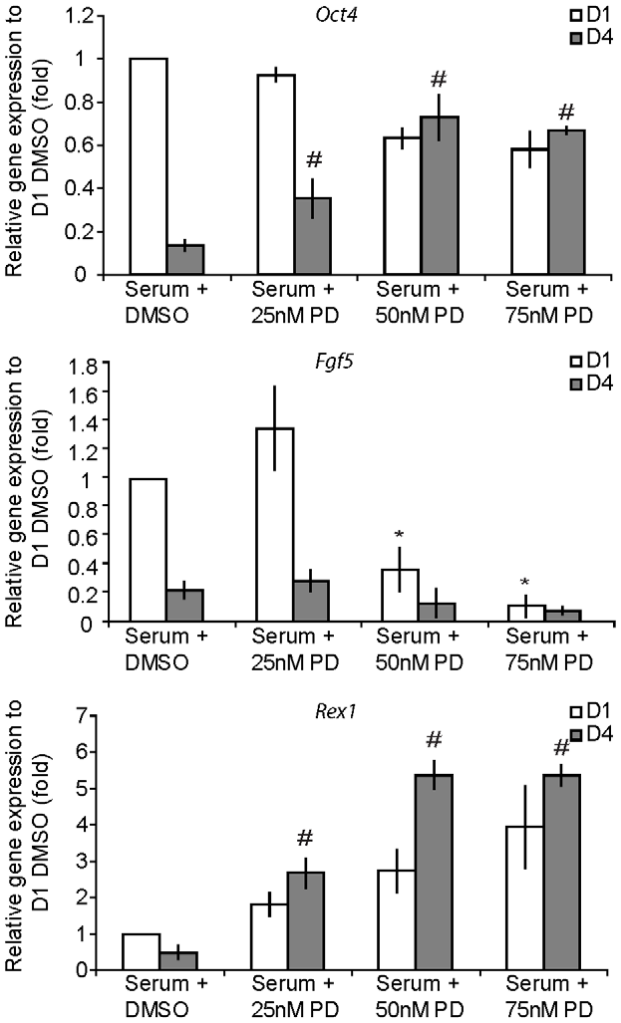
Inhibition of FGF signalling results in increased expression of pluripotent markers. (**A–C**) Quantitative PCR analysis of EPL cells cultured in serum-containing medium in the presence of 25, 50 and 75 nM PD173074 for 1 and 4 days. (**A**) Expression of *Oct4* was found to persist at day 4 in cultures containing PD173074 as compared to DMSO controls (p>0.05). (**B**) Addition of PD173074 at 50 and 75 nM concentrations resulted in a significant decrease in *Fgf5* expression after 1 day of treatment. (**C**) D4 cells cultured in the presence of 25, 50 and 75 nM PD173074 showed significant increases of *Rex1* as compared to D4 DMSO controls. All data were analysed relative to GAPDH and normalised to D1 serum + DMSO controls. Error bars represent SEM. * indicates a significant decrease when compared to D1 serum + DMSO controls; p<0.05. # indicates a significant increase when compared to D4 serum + DMSO control s; p<0.05. n = 3.

FGF 2 has been previously reported to maintain pluripotency in human ES cells [55]–[57], suggesting a role in pluripotent cell regulation. The effect of blocking FGF signalling on the regulation of pluripotence during EPL cell differentiation was investigated. Cells differentiated in PD173074 (25, 50 and 75 nM) were analysed on days 1 and 4 for the expression of pluripotent (*Oct4*), ICM (*Rex1*) and primitive ectoderm (*Fgf5*) markers by qRT-PCR. *Oct4* and *Rex1* expression was significantly higher in cells that had been differentiated for 4 days in the presence of PD173074 when compared with DMSO-containing controls (Fig. 6). Conversely, significantly fewer *Fgf5* transcripts were detected after 24 hours in cells differentiated in PD173074 at 50 and 75 nM when compared to day 1 controls containing DMSO only (Fig. 6); expression of *Fgf5* remained low on day 4 in PD173074 treated cells.

## Discussion

### FGF signalling is required for differentiation to form mesoderm from EPL cells

Previous studies have implicated the involvement of FGF signalling in the formation of a receptive pluripotent intermediate from ES cells [58], mesoderm and endoderm in the primitive streak and in the establishment of the ectoderm lineage [19], [28]. Specifically, FGF signalling has been implicated in the migration of cells through the primitive streak [28] and in neural specification via activation of the ERK signalling pathway [13]. We have exploited a model of the primitive ectoderm, EPL cells, to analyse the effect of FGF signalling in the differentiation of this cell population. Inhibition of endogenous FGF signalling during EPL cell differentiation resulted in the inhibition of mesoderm formation and decreased expression of early primitive streak markers such as *brachyury*. During gastrulation, *brachyury* expression initiates in the epiblast and precedes streak formation [59] suggesting that the first requirement for FGF signalling in gastrulation may be prior to primitive streak formation and lineage specification.

At higher concentrations of inhibitor a total loss of primitive streak markers was observed; the ability of the inhibitor to suppress primitive streak was dose dependent. In another study, Willems and Leyns [34] observed expression of *brachyury* in differentiating ES cells in the presence of FGF inhibition by SU5402. Potentially the concentration of SU5402 used, or the efficiency of this inhibitor, was unable to prevent completely the formation of the primitive streak intermediates and revealed a later requirement for FGF signalling in facilitating EMT.

### FGF signalling is unable to induce or pattern mesoderm

Increasing the concentration of FGFs during EPL cell differentiation, by adding exogenous FGFs 1, 4 or 8, failed to affect the outcomes of differentiation or the gene expression profiles observed during differentiation. Coupled with the requirement for endogenous FGF signalling, this suggests that FGF signalling is required but not sufficient for the formation of the mesoderm lineage from EPL cells. This is consistent with the ability of FGF2 to maintain and not differentiate human ES cells in culture [55], [60]–[62] and the role of FGF signalling in differentiation of ES cells to neural, but not mesoderm lineages, in culture [63]. Neural differentiation was observed from cells cultured in the presence of the FGF signalling inhibitor in the differentiation assays presented here. These cells were also cultured in the presence of serum, a potent source of signalling activity, that we propose will compensate for FGF signalling and enforce differentiation of the cells to the ectoderm. This is consistent with Willems and Leyns [34], who reported a comparable upregulation of the neural marker *Sox1* in ES cells differentiated in the presence of FGF inhibitor and serum.

Alternatively, we considered that FGF signalling may be involved in the specification of mesoderm subtypes during differentiation. The addition of FGFs 4 or 8 in combination with BMP4, however, failed to change the differentiation outcome or gene expression pattern of differentiation. This observation is similar with a previous study demonstrating that FGF2 was unable to synergistically induce mesoderm with BMP4 [64]. Collectively, these observations suggest that FGF signalling was unable to induce, enhance or pattern mesoderm formation from EPL cells in culture.

BMP4 induction of primitive streak intermediates and mesoderm resulted in an increase in *Fgf8*, but not *Fgf4*, expression. FGF signalling has been implicated in several processes in gastrulation, including a role in regulating *E-cadherin* expression and promoting the migration of nascent mesoderm in the primitive streak [28]. The induction of *Fgf8* by BMP4 during primitive streak intermediate and mesoderm formation may serve to ensure all cells are exposed to FGF signalling and susceptible to differentiation. Alternatively, *Fgf8* may play a later role in promoting cell migration in primitive streak intermediates committed to mesoderm.

### A role for FGF signalling prior to lineage specification

Recently, FGF signalling has been reported to be involved in both endoderm and neurectoderm formation [13], [35]. The requirement for FGF signalling in the formation of primitive streak-intermediates shown here, and in neural specification via ERK activation [13] is consistent with a model that places FGF signalling upstream of all germ lineage formation at gastrulation. An increased expression of *Oct4* and *Rex1* was seen after 4 days of EPL cell differentiation in FGF inhibitors when compared to untreated controls. Conversely, in treated populations the expression of *Fgf5* expression was significantly lower when compared to untreated controls. These data suggest that a proportion of the cell population differentiated in the presence of FGF signalling inhibitors retains pluripotency and fails to express high levels of *Fgf5*, implicating FGF signalling in loss of pluripotency and epiblast progression. Coupled with the reduced capability of cells to differentiate to primitive streak-like intermediates, these data suggest that FGF signalling in the epiblast functions to make primitive ectoderm competent to differentiate. This is consistent with recent observations that FGF signalling influences primitive endoderm and epiblast development of ICM cells [65], stabilises the epiblast state in epiSCs [66] and the previously proposed role for ERK signalling in ES cell differentiation [58]. As PD173074 inhibits FGF signalling competitively by acting on FGF receptors, the reduction of *Fgf5* expression may also indicate the presence of positive feedback by FGF signalling on *Fgf5* expression. From these data it is tempting to speculate that increasing levels of *Fgf5* in the primitive ectoderm may reach a point that triggers the loss of pluripotency and adoption of a cell state receptive to inductive signals that function to specify the formation of the germ lineages, thereby ensuring the coordinated and complete differentiation of pluripotent cells during gastrulation.

Using EPL cells as a model system has allowed us to show that FGF signalling is necessary but not sufficient for the formation of the primitive streak lineages *in vitro* and suggest a role for FGF signalling upstream of lineage specification in epiblast maturation prior to gastrulation.

## Materials and Methods

### Verification of biological activities of FGFs 1, 4, 8 and the FGFR inhibitor PD173074

Previous studies have shown a proliferative response in rat lens epithelial cells when treated with 250 ng/mL and 25 ng/mL of FGF1 and FGF2 respectively [67]–[68]. This assay was used to show that the purchased FGFs 1, 4 and 8 (R&D Systems) and the FGF receptor inhibitor PD173074, were biologically active. Lens epithelial explants from day 3 Wistar rats were cultured in the presence of 250 ng/mL of FGF1, 4 and 8 or 250 ng/mL of FGF1 with 25 nM PD173074. After 48 hours in culture, BrdU was added to a final concentration of 150 µg/mL and explants cultured for a further 8 hours. Explants were fixed and stained with anti-BrdU antibodies conjugated with HRP enzymes as described [67]–[68]. Explants treated with the FGFs had an increased BrdU incorporation when compared to untreated controls (Figure S1). In explants treated with both FGF1 and PD173074, the increase in proliferating cells was less than that seen in explants treated with FGF1 alone (Figure S1). These findings indicate that the FGFs and the FGFR specific inhibitor to be used in this study were biologically active.

### Cell culture

ES cell line D3 [31] was used in this study. The culture, maintenance, formation of EPLs from D3 ES cells and formulation of the conditioned media MEDII to form EPL cells were as described previously in [69]. For all experiments, EPL cells were formed within cellular aggregates [70]. Briefly, a single cell suspension of D3 ES cells was seeded at 1×10^5^ cells/mL in 50% MEDII [69]. Aggregates were divided 1 in 2 and re-fed on day 3. All treatments were initiated using aggregates that had been maintained for 3 days in MEDII.

### Differentiation assays

EPL cell aggregates were transferred to differentiation medium [69] or chemically defined medium (50% DMEM, 50% Hams F12 (Invitrogen) supplemented with 1 x insulin transferring sodium selenite (Sigma), 0.1 mM β-mercaptoethanol (β-ME; Sigma), 50 u/mL penicillin (Chemicon), 50 µg/mL streptomycin (Chemicon)) supplemented with FGFs, BMP4 and/or PD173074, as described in the text, and cultured for a further 3 days. At this point, aggregates were transferred from suspension culture to adherent culture. Aggregates were placed individually into 2 mL tissue culture wells which had been pre-treated with gelatin and contained chemically defined medium [69]. Aggregates were scored on day 6 for the presence of three morphologically distinctive cell types, beating cardiomyocytes, visible red blood or neural extensions as described previously [41]–[42]; cells with neural projections have been shown previously to express βIII-tubulin and NeuN [70]. Generally, for each experimental condition 24 individual wells with randomly selected cellular aggregates were scored and experiments were repeated a minimum of three times. Data collected was analysed statistically with a two-tailed student's t-test.

### Gene expression analysis

#### RT-PCR

Total cytoplasmic RNA was extracted using Trizol reagent (Invitrogen). cDNA was synthesised using M-MLV Reverse Transcriptase (Promega). For all PCR reactions in this study, reaction tubes contained a final volume of 25 µL containing 1 ng/µL each of forward and reverse primers, 1 x concentration of GoTaq® Green Master Mix (Promega) and cDNA. Samples were heated to 95°C for 2 minutes before cycled according to table 1 at 95°C for 30 s, 60°C for 30 s and 72°C for 30 s before a 5 minute final extension step at 72°C on an MJ Research thermocycler. A list of primer sequences, number of amplification cycles used and expected amplicon size are listed in Table 1.

#### qRT-PCR

RNA was isolated and cDNA synthesised as for RT PCR. qPCR reaction mix consisted of 1X Absolute blue QPCR SYBR Green Mix (Thermo Scientific) with 200 nM of forward and reverse primers (*GAPDH* & *Fgf8*; Table 1). Reactions were amplified on an MJ research thermocycler with a Chromo4 Continuous Fluorescence Detection system (MJ Research). The raw data was analysed using the Q-Gene software package [71]–[72].

#### Immunocytochemistry

EPL cells aggregates were cultured as described in the differentiation assays but were allowed to adhere to 0.2% gelatin treated glass coverslips. The adherent cells were fixed with 4% paraformaldehyde 6 days after seeding onto coverslips and washed with PBS. Fixed cells were permeabilised with 0.25% Triton X-100 in PBS for 20 minutes. Coverslips were blocked with 0.5% Tween20, 1% FBS in PBS for 30 minutes. Primary antibody staining was performed with mouse monoclonal anti βIII-tubulin (Sigma, T8578) and anti cardiac troponin 1 (Abcam, ab19615) overnight in a humidified chamber. Secondary antibody staining was performed with Alexa Fluor® 488 rabbit anti-mouse IgG (Invitrogen, A11059).

## Supporting Information

Figure S1 FGF 1, 4 or 8 was able to induce proliferation in rat lens epithelial cells. (A-E): Rat lens epithelial explants treated with (A) 250 ng/ml FGF1, (B) 250 ng/ml FGF4, (C) 250 ng/ml FGF8, (D) 250 ng/ml FGF1 with 25 nM PD173074 or (E) untreated. (F): Average of 6 randomly counted 200-by-200 micron quadrants across all treatments and control explants. A two-tailed student's t-test demonstrates a significant increase in BrdU incorporation in all treatments when compared to untreated controls. FGF4 and FGF8 were less effective in inducing cell proliferation (32% and 30% respectively) than FGF1 (44%). Inclusion of 25 nM PD173074 significantly inhibited (p<0.05) proliferation induced by 250 ng/ml FGF1. * indicates a significant increase when compared to controls where p<0.05. # indicates a significant decrease when compared to 250 ng/ml Fgf1 where p<0.05.
(5.34 MB TIF)
