# Supplementary figures and images for: A Requirement for FGF Signalling in the Formation of Primitive Streak-Like Intermediates from Primitive Ectoderm in Culture
Source: PLoS One. 2010 Sep 3;5(9):e12555. doi: 10.1371/journal.pone.0012555 (PMC2933233; doi:10.1371/journal.pone.0012555)

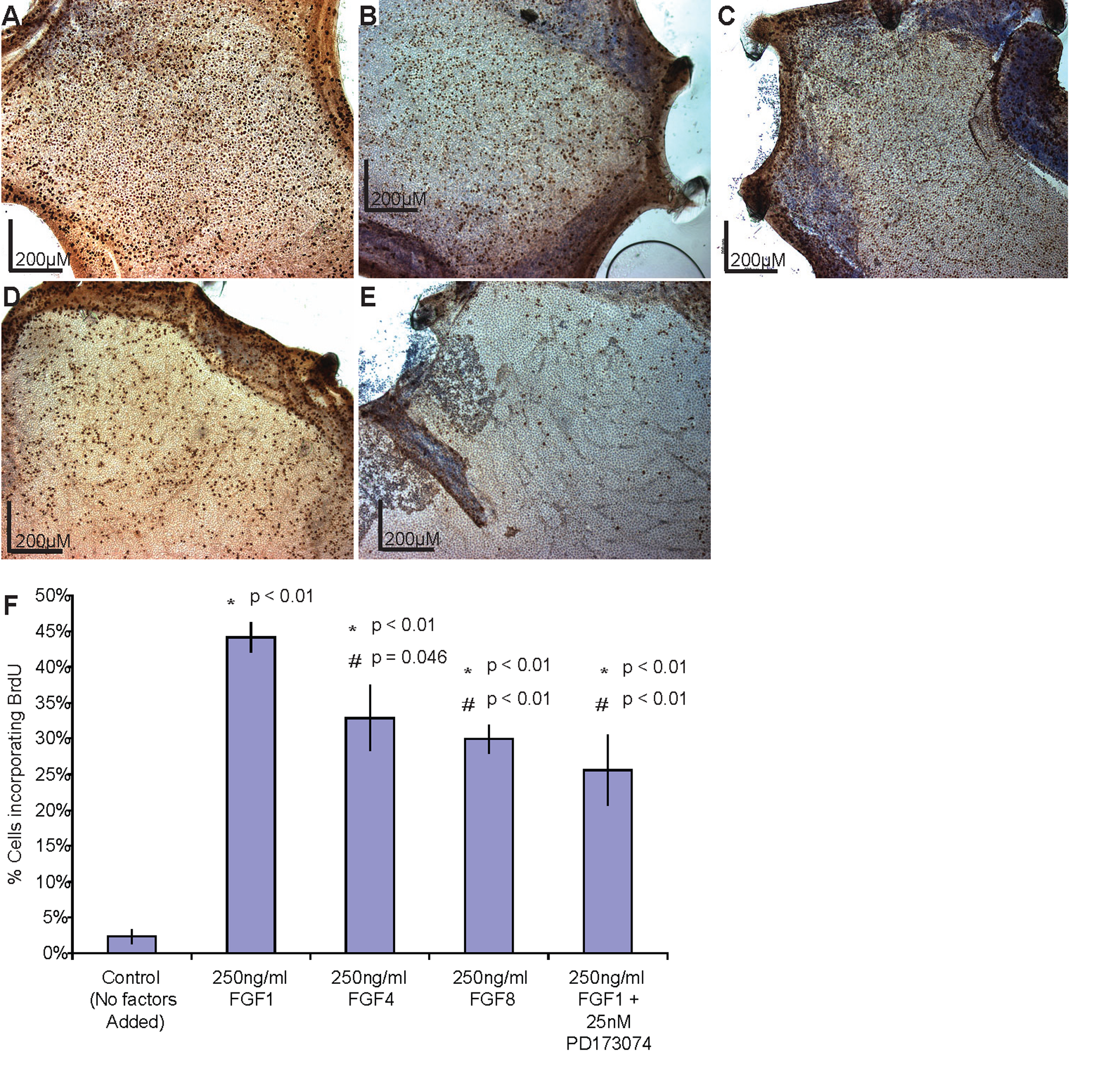

Supplement: Figure S1 — FGF 1, 4 or 8 was able to induce proliferation in rat lens epithelial cells. (A-E): Rat lens epithelial explants treated with (A) 250 ng/ml FGF1, (B) 250 ng/ml FGF4, (C) 250 ng/ml FGF8, (D) 250 ng/ml FGF1 with 25 nM PD173074 or (E) untreated. (F): Average of 6 randomly counted 200-by-200 micron quadrants across all treatments and control explants. A two-tailed student's t-test demonstrates a significant increase in BrdU incorporation in all treatments when compared to untreated controls. FGF4 and FGF8 were less effective in inducing cell proliferation (32% and 30% respectively) than FGF1 (44%). Inclusion of 25 nM PD173074 significantly inhibited (p<0.05) proliferation induced by 250 ng/ml FGF1. * indicates a significant increase when compared to controls where p<0.05. # indicates a significant decrease when compared to 250 ng/ml Fgf1 where p<0.05. (5.34 MB TIF) [file pone.0012555.s001.tif]
